# The Predictive and Prognostic Factors in Patients with Gastric Cancer Accompanied by Gastric Outlet Obstruction

**DOI:** 10.1155/2020/6529563

**Published:** 2020-07-25

**Authors:** Hongliang Zu, Huiling Wang, Chunfeng Li, Wendian Zhu, Yingwei Xue

**Affiliations:** ^1^Department of Gastroenterologic Surgery, The First People's Hospital of Zhaoqing, China; ^2^Department of ICU, The First People's Hospital of Zhaoqing, China; ^3^Department of Gastroenterologic Surgery, Affiliated Tumor Hospital of Harbin Medical University, China

## Abstract

**Purpose:**

This study is aimed at evaluating the clinicopathological features and prognostic significance of gastric outlet obstruction (GOO) in patients with distal gastric cancer.

**Methods:**

A retrospective review of 1564 individuals with distal gastric cancer from 2002 to 2010 was performed. In total, 157 patients had GOO. The clinicopathological features of the patients with GOO were compared with those of the patients without GOO. A Kaplan-Meier survival analysis and Cox proportional hazard model were used to assess the overall survival.

**Results:**

The patients with distal gastric cancer with GOO generally presented more aggressive pathologic features, a poorer nutritional status, more duodenal infiltration, and peritoneal dissemination than those with cancer without GOO. In the univariate analysis, curability, GOO, age, prealbumin, albumin, hemoglobin (Hb), the tumor size, the macroscopic type, lymph node metastasis, and the depth of invasion had a statistically significant influence on prognosis. The multivariate analysis showed that curability, GOO, the tumor size, lymph node metastasis, and the depth of invasion were independent prognostic factors.

**Conclusions:**

Gastric cancer with GOO exhibits aggressive biological features and has poor outcomes. The multivariate analysis showed that curability, GOO, the tumor size, lymph node metastasis, and the depth of invasion were independent prognostic factors. The gastric outlet status should be considered in the selection of surgical treatment methods for patients with gastric cancer.

## 1. Background

Despite the steady decline in its incidence rate and mortality in recent years, more than 1.22 million incident cases of stomach cancer occurred worldwide in 2017, and nearly 865000 people died of stomach cancer. In East Asia, China alone had nearly half of the global incident cases in 2017, and approximately two-thirds of the cases died of stomach cancer [1]. Gastric cancer with GOO most often manifests in the distal third of the stomach and is usually found in individuals with advanced disease. Indeed, GOO and hemorrhage are surgical indications for advanced gastric cancer, and it is often necessary for the surgeon to intervene or relieve clinical symptoms to prolong the patient's survival. Different treatment methods are currently utilized. Surgical treatments include radical resection, palliative resection, and gastrojejunostomy, while nonoperative treatments include endoscopic stent therapy and combined chemotherapy. These approaches [2, 3] can alleviate complications, such as bleeding and obstruction, in patents with GOO. The effectiveness of surgery has been discussed [4], and it has been suggested that the morbidity and mortality of radical surgery can be acceptable in patients with GOO [5], while another study showed that the presence of GOO can predict increased postoperative morbidity following D2 gastrectomy for gastric cancer [6]. The aim of our study was to compare the differences in clinicopathological features between patients with and without GOO and explore the prognosis of the former to provide a basis for clinical treatment.

## 2. Patients and Methods

### 2.1. Patient Selection and Study Design

Between 2002 and 2010, 1564 patients with histologically proven primary gastric adenocarcinoma underwent gastrectomy at the Department of Surgical Gastroenterology, Affiliated Tumor Hospital of Harbin Medical University, Harbin, China. This retrospective study was approved by the Ethics Committee of Harbin Medical University. The clinicopathological data were retrospectively obtained from the medical records of each patient and an electronic database. All patients were routinely followed every three months for the first two years, every 6 months for the following 3 years, and once a year thereafter. The patient follow-up lasted until death or the cutoff date of January 1, 2015. For the patients who survived, the data were censored at the date of the final contact. Only cases in which patients died of gastric cancer were classified as tumor-related deaths. None of the included patients received preoperative chemotherapy or radiotherapy. All enrolled patients had symptoms associated with GOO, including nausea, vomiting, or the inability to consume a regular diet. The diagnosis of GOO [7] was based on the findings of upper endoscopy or a radiologic assessment, including abdominal computed tomography (CT) or upper digestive tract radiography, and the abovementioned symptoms of GOO. While preparing for operation in the patients with GOO, a nasogastric tube was used to decompress the stomach and correct the imbalance between fluids and electrolytes. Gastric patients with acute obstruction who underwent emergency surgery were excluded. The hospital's database was queried to identify cases of cancer arising in the distal third of the stomach, which was defined by subdividing the lesser and greater curvatures into the following three equal lengths as described in the Japanese Classification of Gastric Cancer [7]: proximal, middle, and distal. Extended lymph node dissection was performed routinely according to the guidelines of the Japanese Research Society for Gastric Cancer and included the lymph nodes along the perigastric region, celiac axis, hepatoduodenal ligament, and retropancreatic region [8]. The following clinicopathological data were collected: sex (male or female); age (mean, SD); tumor size (mean, SD); Hb (mean, SD); prealbumin (mean, SD); albumin (mean, SD); macroscopic type (Borrmann EGC, I, II, III, IV, or X); degree of differentiation (well differentiated, moderately differentiated, poorly differentiated, mucinous carcinoma, or signet ring cell carcinoma; if there were two or more histological types, the histological type was defined by the predominant type in the tumor); depth of tumor invasion (T1: tumor invaded the mucosa or submucosa layer; T2: tumor invaded the muscular layer or the subserosa; T3: tumor invaded the subserosa; T4a: tumor invaded the serosa or penetrating serosa; or T4b: tumor invaded adjacent organs); and 7th American Joint Committee on Cancer (AJCC) lymph node status (N0, N1, N2, N3a, or N3b). Regarding the curability of the operation (curative or noncurative), surgery was deemed curative when there was no residual tumor (R0 resection); otherwise, surgery was considered noncurative (R1 or R2) [9]. Laparotomy and bypass (gastrojejunostomy) procedures were also recorded in the scope of our study. Moreover, we evaluated the involvement of adjacent organs, including the duodenum, pancreas, liver, greater omentum, transverse mesentery, and peritoneum. A multivariate analysis using a logistic regression model was performed to identify the factors associated with GOO.

### 2.2. Statistics

Chi-squared and Fisher exact tests were used to analyze the associations between the categorical variables. The survival data were estimated using the Kaplan-Meier method, and the log-rank test was employed to compare the differences in survival between the gastric cancer patients with and without GOO. A multivariate analysis of the prognostic factors related to overall survival was carried out using Cox proportional hazard models. The criterion for statistical significance was *p* < 0.05. All data analyses were performed using SPSS for Windows, Version 22.0 software (SPSS, Inc., Chicago, IL, USA).

## 3. Results

### 3.1. Clinicopathological Features

The clinicopathological features of the patients with and without GOO were compared (Table 1). The analysis of the clinicopathological features revealed no differences related to age, although the patients with GOO were slightly older than those without GOO (60.21 years vs. 56.24 years). The tumor size in the patients with GOO was larger than that in the patients without GOO (6.44 cm vs. 5.63 cm, *p* = 0.006). Compared to the patients without GOO, those with GOO predominantly exhibited Borrmann type IV gastric carcinoma (12.7% vs. 9.2%), deeper tumor invasion (T4a, 43.9% vs. 37.2%; T4b, 40.1% vs. 28.1%; *p* ≤ 0.001), and more lymph node metastasis (N3a, 22.3% vs. 10.3%; N3b, 15.9% vs. 2.7%; *p* ≤ 0.001). There was no difference in the Hb and albumin levels or cell differentiation between those with and without GOO. The prealbumin level in the patients with GOO was lower than that in those without GOO (223.14 mg/L vs. 271.60 mg/L, respectively). Moreover, the patients without GOO experienced a higher cure rate after surgery than the patients with GOO (73.7% vs. 63.7%). Accordingly, there were more cases of palliative resection (22.9% vs. 16%) and gastrojejunostomy (12.7% vs. 7.7%) among the patients with than without GOO. Furthermore, the patients with GOO presented more peritoneal migration (11.5% vs. 5.4%, *p* = 0.007) than the patients without GOO. Regarding the involvement of neighboring organs (Table 2), duodenal invasion showed the most significant difference between the patients with and without GOO (19.7% vs. 7.2%), whereas there was no difference in the invasion of the pancreas, greater omentum, liver, or transverse mesentery. According to the multivariate analysis using a logistic regression model, the factors distinguishing the patients with GOO from those without GOO were lymph node metastasis, the depth of invasion, the prealbumin level, and the Borrmann type (Table 3).

### 3.2. Univariate and Multivariate Survival Analyses

**Figure 1:**
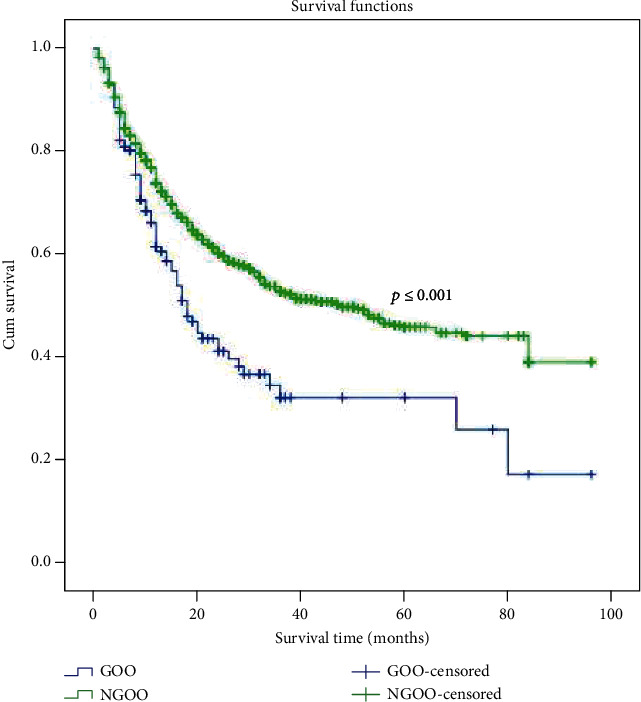
Kaplan-Meier survival curves for 1564 gastrectomy patients according to GOO. The prognosis of gastric cancer patients with GOO was poorer than that of patients without GOO (*p* ≤ 0.001). The five-year survival rate was 25.8% and 45.9% among patients with and without GOO, respectively.

**Figure 2:**
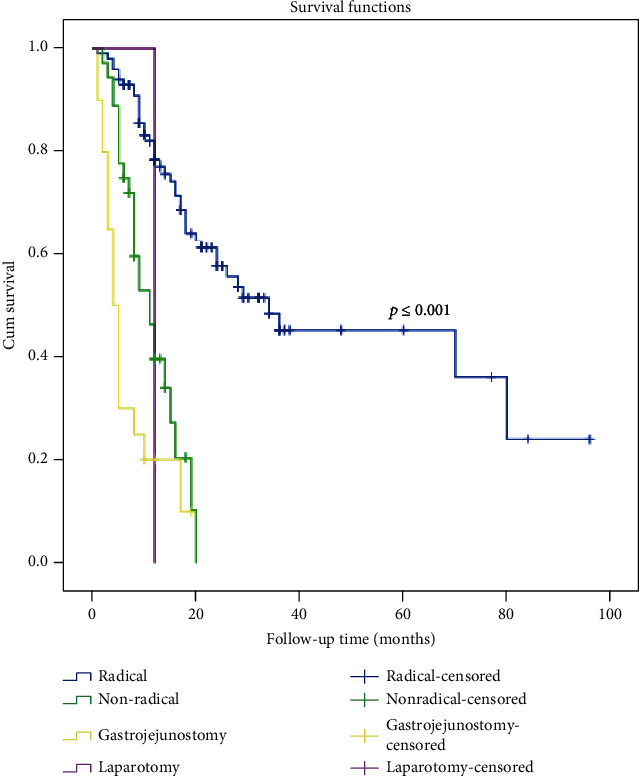
Survival curves for the 157 gastric cancer patients with GOO according to surgical procedure (*p* ≤ 0.001).

To identify the prognostic factors associated with overall survival, univariate and multivariate analyses were used to assess the relevant clinicopathological variables (Tables 4 and 5). The univariate survival analysis indicated a significantly lower 5-year survival rate in the patients with GOO compared with that in those without GOO (25.8% vs. 45.9%, Figure 1). In addition to the gastric outlet status, the significant prognostic factors of survival included age; the tumor size; the prealbumin, albumin, and Hb levels; the Borrmann type; pT; pN; and curability (Table 4). A Cox proportional hazards model revealed that GOO, the tumor size, lymph node metastasis, the depth of invasion, and curability were independent prognostic factors (as shown in Table 5). Moreover, the univariate survival analysis showed that radical resection has a survival benefit over palliative resection or gastrojejunostomy in patients with GOO (Figure 2, *p* ≤ 0.001), and 36.3% (57 in 157) of patients underwent noncurative surgery.

## 4. Discussion

In this retrospective study, we examined the clinicopathological features of patients with distal gastric cancer and the corresponding prognostic significance. The patients with GOO presented a lower prealbumin level, a larger tumor size, deeper cancer invasion, and more lymph node metastasis than the patients without GOO. In our previous study, a larger tumor size was an independent prognostic factor [10]. Chen et al. [11] also revealed that patients with GOO had deeper cancer invasion and more lymph node metastasis than the patients without GOO. The T and N stages are recognized as prognostic factors in gastric cancer. Moreover, the patients with GOO predominantly showed Borrmann type IV gastric carcinoma, which is consistent with the findings reported by Park et al. [5], who reported a higher incidence of Borrmann type IV gastric carcinoma among patients with GOO than those without GOO. This finding may be related to local infiltration, which is likely to cause obstruction.

Moreover, the prognosis of the gastric cancer patients with GOO was poorer than that of those without GOO, and the gastric outlet status was an independent prognostic factor. This result is consistent with the findings reported by Park et al. [5]. Chen et al. [11] also found that patients with GOO had a poorer prognosis than those without GOO, but GOO was not an independent prognostic factor. GOO may be highly correlated with other correlative factors, affecting the role of obstructive factors in the multivariate analysis.

The usual metastatic process of gastric cancer involves local infiltration, lymph node metastasis, and hematogenous metastasis. Gastric cancer with GOO often involves the infiltration of adjacent organs. In this study, the duodenum showed the most infiltration, followed by the pancreas and transverse mesentery; in all cases, the extent of infiltration was related to anatomy. Moreover, GOO was more often accompanied by peritoneal metastasis. This finding is consistent with Sunil et al.'s study [6], which found that GOO most commonly involved direct invasion, followed by peritoneal dissemination and lymph node metastasis with a high incidence of invasion of the pancreas. Ohta et al. [12] reported that gastric cancer with duodenal invasion had a significantly higher incidence of pyloric stenosis-related symptoms than that without duodenal invasion, further verifying the interrelationship between GOO and duodenal invasion. In addition, we found a preponderance of Borrmann type IV disease and larger tumors in the patients with GOO, which may contribute to invasion and lead to peritoneal metastasis.

We found that the prealbumin level in the patients with GOO was lower than that in those without GOO. This finding suggests that prealbumin is more representative of the nutritional status of patients. In addition, the prealbumin level was an independent prognostic factor in the GOO patients. Compared to albumin, prealbumin is affected earlier by acute variations in the protein balance and responds to nutritional support faster [13]. Several studies have shown that prealbumin can be used as a single parameter to evaluate protein energy malnutrition, even for outcomes related to postsurgical status and cancer recurrence [14, 15]. In addition, the prealbumin level at admission was found to be an independent prognostic factor for gastric cancer in our previous study [16]. Our study also demonstrated that the factors affecting the gastric outlet status were lymph node metastasis, the depth of invasion, the prealbumin level, and the Borrmann type in the multivariate logistic analysis. It is necessary to acknowledge the importance of prealbumin when assessing the nutritional status of gastric cancer patients, especially those with GOO.

Our study shows that radical resection provides a notable survival benefit over palliative resection or gastrojejunostomy in patients with GOO, and over one-third of the patients underwent noncurative surgery. Sunil et al. [6] reported that over two-thirds of patients with pyloric stenosis underwent either noncurative or no resection; thus, it was difficult to evaluate the effect of curative resection. The choice of treatment mode for gastric carcinoma with GOO should be individualized. One study showed that the WHO performance score was a significant prognostic factor, and those researchers recommended gastrojejunostomy for patients with a WHO score of 0-1 and stent placement for those with a WHO score of 3-4 [17]. Keränen et al. [4] found that endoscopic stenting resulted in faster improvement in oral intake and symptom relief and a shorter hospital stay in gastric cancer patients with symptoms of GOO. Neoadjuvant chemotherapy can potentially reduce the tumor volume and improve curative opportunities [18, 19]. In the NCCN guide 2018 (class I recommendation), preoperative adjuvant chemotherapy combined with postoperative adjuvant treatment mode is indicated as the preferred treatment mode for patients with expected resectable advanced gastric cancer (≥cT2N0) [20]. However, in the Japanese JGCA guidelines, neoadjuvant chemotherapy or neoadjuvant radiotherapy and chemotherapy are not recommended as the routine treatment and are still defined as an experimental treatment [21]. Few published studies [22] have indicated the potential feasibility of preoperative chemotherapy for gastric cancer patients with GOO. Hyperthermic intraoperative intraperitoneal chemotherapy (HIPEC) is considered an effective and attractive method for the treatment of peritoneal malignancies. Cianci et al. [23] showed that HIPEC for peritoneal malignancies using a new hybrid CO_2_ system is feasible and yields good perioperative outcomes. GOO was more often accompanied by peritoneal metastasis, and in these patients, HIPEC was carried out first. In the future, we will first apply gastrojejunostomy or endoscopic stenting, followed by neoadjuvant chemotherapy or HIPEC and then surgery to improve the survival of distal gastric cancer patients with GOO.

Our study lacked data regarding the administration of preoperative chemotherapy and postoperative adjuvant chemotherapy as there was uncertainty regarding the extent of systemic adjuvant postoperative chemotherapy for most patients. Another limitation was the lack of data regarding postoperative complications. Complications may influence the postoperative performance status and survival of patients. Future prospective clinical studies should assess the survival benefit of postoperative chemotherapy in patients with GOO and follow their condition and complications.

## 5. Conclusion

Gastric cancer with GOO exhibits aggressive biological features and has poor outcomes. The multivariate analysis showed that curability, GOO, the tumor size, lymph node metastasis, and the depth of invasion were independent prognostic factors. The gastric outlet status should be considered when selecting surgical treatment methods. Considering the poor prognosis of patients with pyloric obstruction and that radical resection provides a notable survival benefit over palliative resection or gastrojejunostomy, we can take some active treatment measures. Laparoscopic staging can be performed first, followed by HIPEC or neoadjuvant chemotherapy in patients who cannot undergo radical operation, and radical surgery could be performed after the tumor shrinks in the future.

## Figures and Tables

**Table 1:** Clinicopathological features and treatment-related factors of the patients with and without GOO groups.

Variables	GOO (157)	Without GOO (1407)	*p*/*t* value
Gender			0.224
Male	120/76.4%	1011/71.9%	
Female	37/23.6%	396/28.1%	
Age (years) mean, SD^※^	60.21 ± 11.15	56.24 ± 11.82	≤0.001
Tumor size (cm) mean, SD	6.44 ± 2.64	5.63 ± 3.59	0.006
Hb^#^ (g/L), mean, SD	125.34 ± 25.63	125.36 ± 30.57	0.996
Prealbumin (mg/L) mean, SD	223.14 ± 65.21	271.60 ± 88.53	≤0.001
Albumin (g/L) mean, SD	41.92 ± 25.89	41.29 ± 28.40	0.79
Stage N^+^			≤0.001
N0	33/21%	382/27.1%	
N1	20/12.7%	334/23.7%	
N2	19/12.1%	367/26.1%	
N3a	35/22.3%	145/10.3%	
N3b	25/15.9%	38/2.7%	
NX	25/15.9%	141/10.0%	
Stage T^∗^			≤0.001
T1	3/1.9%	138/9.8%	
T2	20/12.7%	305/21.7%	
T3	2/1.3%	45/3.2%	
T4a	69/43.9%	524/37.2%	
T4b	63/40.1%	395/28.1%	
Surgery			0.005
Radical resection	100/63.7%	1037/73.7%	
Nonradical resection	36/22.9%	225/16.0%	
Gastrojejunostomy	20/12.7%	108/7.7%	
Laparotomy	1/0.6%	37/2.6%	
Macroscopic type			0.002
EGC^@^	1/0.6%	59/4.3%	
Borrmann I	5/3.2%	120/8.7%	
Borrmann II	12/7.6%	114/8.3%	
Borrmann III	92/58.6%	819/59.5%	
Borrmann IV	20/12.7%	126/9.2%	
*x*	27/17.2%	139/10.1%	
Differentiation			0.181
Well	7/4.5%	44/3.1%	
Moderate	45/28.7%	452/32.1%	
Poor	81/51.6%	652/46.3%	
Mucinous	8/5.1%	71/5.0%	
Signet	11/7.0%	73/5.2%	
*x*	5/3.2%	115/8.2%	
Peritoneal metastasis			0.007
No	139/88.5%	1331/94.6%	
Yes	18/11.5%	76/5.4%	

^∗^T1 tumor has invaded the mucosa or submucosa layer; T2 tumor has invaded the muscular layer; T3 tumor has invaded the subserosa; T4a: tumor has invaded the serosa or penetrating serosa; T4b: tumor invaded the adjacent organs; ^+^N0 no regional lymph node metastasis; N1 1-2 regional lymph node metastasis; N2 3-6 regional lymph node metastasis; and N3a 7-15 regional lymph node metastasis; N3b ≥15 regional lymph node metastasis. ^※^SD standard deviation; ^#^Hb hemoglobin; ^@^EGC early gastric cancer; *x* unknown type.

**Table 2:** The invasion of adjacent organs in patients with and without GOO.

Invasion of adjacent organ	GOO (157)	Without GOO (1407)	*p*/*t* value
Pancreas			0.072
No	117/74.5%	1138/80.9%	
Yes	40/25.5%	269/19.1%	
Greater omentum			0.522
No	153/97.5%	1366/97.1%	
Yes	4/2.5%	41/2.9%	
Duodenum			≤0.001
No	126/80.3%	1306/92.8%	
Yes	31/19.7%	101/7.2%	
Transverse mesentery			0.91
No	131/83.4%	1177/83.7%	
Yes	26/16.6%	230/16.3%	
Liver			0.562
No	153/97.5%	1525/97.5%	
Yes	4/2.5%	35/2.5%	

**Table 3:** Multivariate logistic analysis of the with and without GOO groups for related factors.

Variables	*χ* ^2^	*p*
Stage N	60.87	≤0.001
Stage T	10.846	0.028
Prealbumin	42.706	≤0.001
Macroscopic type	21.238	0.001

**Table 4:** Univariate survival analysis of related clinicopathological variables (*n* = 1564).

Variables	*χ* ^2^	*p*
Gender	0.698	0.403
Age	80.632	0.047
Radical or not	744.031	≤0.001
Prealbumin	284.135	≤0.001
Albumin	538.733	≤0.001
Hb	264.463	≤0.001
Tumor size	445.445	≤0.001
Stage N	241.98	≤0.001
Stage T	211.70	≤0.001
GOO	17.909	≤0.001
Differentiation	4.121	0.532
Macroscopic type	12.962	0.024

**Table 5:** Multivariate survival analysis in 1564 gastric cancer patients with and without GOO.

Variables	*χ* ^2^	*p*	Hazard ratio (95% CI)
Radical or not	213.015	≤0.001	0.081-0.184
Tumor size	10.754	0.001	1.016-1.064
T	38.344	≤0.001	0.841-1.602
N	50.285	≤0.001	0.118-0.408
GOO	3.879	0.049	1.001-1.633

## Data Availability

This study data is from the “Department of Gastroenterologic Surgery, Affiliated Tumor Hospital of Harbin Medical University, Harbin (150040), China,” and from patients with gastric cancer who underwent resection for gastric cancer at the Affiliated Tumor Hospital of Harbin Medical University, from January 2002 to December 2010. Raw data cannot be provided for personal and commercial purpose.

## Abbreviations

SD: Standard deviation
Hb: Hemoglobin
EGC: Early gastric cancer
*x*: Unknown type
GOO: Gastric outlet obstruction
AJCC: American Joint Committee on Cancer
WHO: World Health Organization.
